# Efficient simulation of clinical target response surfaces

**DOI:** 10.1002/psp4.12779

**Published:** 2022-03-11

**Authors:** Daniel Lill, Anne Kümmel, Venelin Mitov, Daniel Kaschek, Nathalie Gobeau, Henning Schmidt, Jens Timmer

**Affiliations:** ^1^ IntiQuan GmbH Basel Switzerland; ^2^ Institute of Physics University of Freiburg Freiburg Germany; ^3^ Medicines for Malaria Venture Geneva Switzerland; ^4^ Centre for Integrative Biological Signalling Studies (CIBSS) University of Freiburg Freiburg Germany; ^5^ Freiburg Center for Data Analysis and Modelling (FDM) University of Freiburg Freiburg Germany

## Abstract

Simulation of combination therapies is challenging due to computational complexity. Either a simple model is used to simulate the response for many combinations of concentration to generate a response surface but parameter variability and uncertainty are neglected and the concentrations are constant—the link to the doses to be administered is difficult to make—or a population pharmacokinetic/pharmacodynamic model is used to predict the response to combination therapy in a clinical trial taking into account the time‐varying concentration profile, interindividual variability (IIV), and parameter uncertainty but simulations are limited to only a few selected doses. We devised new algorithms to efficiently search for the combination doses that achieve a predefined efficacy target while taking into account the IIV and parameter uncertainty. The result of this method is a response surface of confidence levels, indicating for all dose combinations the likelihood of reaching the specified efficacy target. We highlight the importance to simulate across a population rather than focus on an individual. Finally, we provide examples of potential applications, such as informing experimental design.

**Study Highlights**

**WHAT IS THE CURRENT KNOWLEDGE ON THE TOPIC?**

Population simulation of PKPD models and response surface analysis currently co‐exist as distinct methods for model‐based combination therapy assessment. Each method is tailored to address specific questions but neglects important aspects of the other method.

**WHAT QUESTION DID THIS STUDY ADDRESS?**

The two challenges of applying population simulation and response surface analysis jointly are: How to informatively summarize the output of population simulation such that it can be interpreted in a response surface, and how to perform the computations efficiently?

**WHAT DOES THIS STUDY ADD TO OUR KNOWLEDGE?**

Population simulation results can be informatively summarized by the confidence level to reach a prespecified efficacy target. We propose two algorithms to compute confidence level response surfaces with a fraction of the computing time required by brute force.

**HOW MIGHT THIS CHANGE DRUG DISCOVERY, DEVELOPMENT, AND/OR THERAPEUTICS?**

We provide a general framework of how to incorporate variability and parameter uncertainty into response surface analysis and show how response surface analysis has an application in a clinical context.

## INTRODUCTION

Combination therapies of two or more partner drugs are the standard in many indications, such as malaria,^1^ bacterial infections,^2^, ^3^ or cancer.^4^, ^5^ Different benefits arise from the use of combinations. For example, the emergence of resistance is slowed down or existing resistances are overcome by tackling the disease on independent mechanisms of action^6^, ^7^ or the joint effect may be reaching the therapeutic target at overall lower doses, reducing treatment cost and toxicity. Population pharmacokinetic/pharmacodynamic (PK/PD) models of combination therapies are an emerging field. Applications range from semimechanistic drug‐drug interaction modeling^3^, ^8^, ^9^ to dose optimization.^10^, ^11^, ^12^ Especially the latter application, as well as model‐based prediction of clinical trial results, rely on model simulations which are impeded by the combinatorial complexity of combination therapies.

The two main approaches for simulation‐based combination therapy assessment are: (1) response surface and isobolographic analysis,^13^, ^14^ and (2) population simulation,^15^ both suffering from shortcomings. For the former, response surfaces and isoboles are useful tools to establish a comprehensive overview of the treatment and to explore drug‐drug interactions. However, they usually are evaluated for concentration‐response rather than dose‐response relationships,^4^, ^9^, ^16^ neglecting effects governed by PKs and PDs. Furthermore, they are calculated only for a unique set of parameters, neglecting both interindividual variability (IIV) and parameter uncertainty. This hampers their application in the clinical setting. The second method, population simulation using nonlinear mixed effects (NLMEs) PK/PD models, accounts for PKs and PDs and allows the calculation of clinical efficacy end points, including their confidence intervals. However, it is often performed only for relatively few doses.^3^, ^8^, ^16^, ^17^ Global trends and dosing opportunities might thus be overlooked.

We propose fast new algorithms based on both population simulation and response surface analysis to fully characterize the dose‐response of combination therapies taking into account PKs, PDs, parameter uncertainty, and IIV. By focusing on a predefined efficacy target, like responder rate of 95%, the number of simulations can be reduced down to 10% of a brute force approach. The output is a confidence level response surface and confidence level isoboles which show all the minimum combination doses that achieve the target of interest, here, 95% responder rate, at any given confidence level, for instance, at a confidence level of 80%. We apply the methodology to malaria and antibiotics research as examples and discuss how these simulations can inform further the drug‐development process.

## METHODS

**FIGURE 1:**
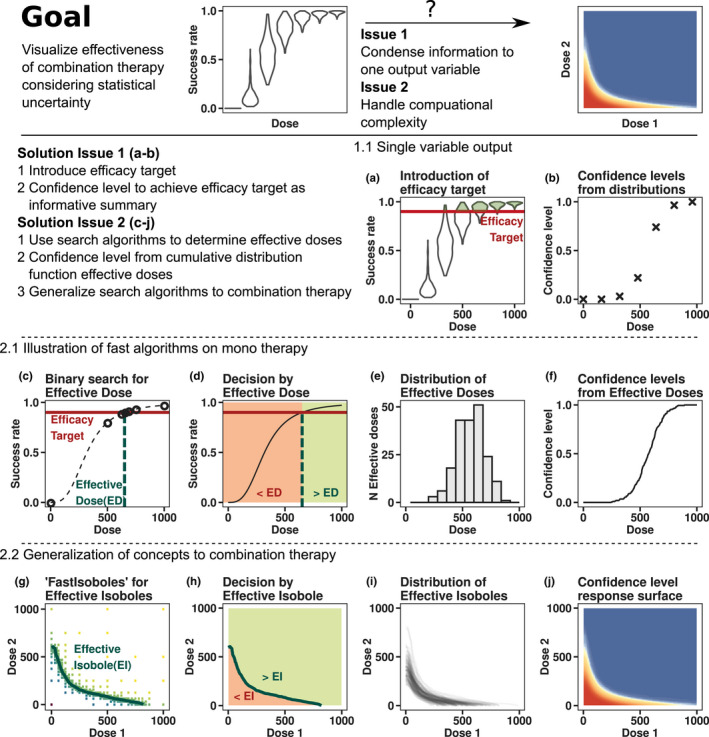
Overview of methods. (a) Success rate distributions resulting from parameter uncertainty, simulated at different doses. The efficacy target, a success rate of 90%, is highlighted as the red horizontal line. Shaded green areas of violin plots correspond to the fraction of populations meeting the efficacy target criterion. (b) Confidence level of reaching the efficacy target at a given dose. The confidence level corresponds to the green shaded areas in panel a. The confidence level is known exactly at only seven doses. (c) The dose‐success rate relationship for one population is shown as the dashed black line. The effective dose (dashed green line) indicates where success rates achieve the efficacy target (red horizontal line). Circles denote the iterations of a binary search algorithm which, in this case, finds the effective dose within five iterations, amounting to seven function evaluations. (d) The effective dose allows to classify any dose into non‐efficacious (red area) or efficacious (green area) via a simple “less than” operation. (e) Distribution of effective doses resulting from parameter uncertainty. (f) Confidence levels as cumulative distribution function of effective doses. The confidence level is known exactly at a much higher resolution but the computations took as many function evaluations as in panel b. (g) Newly developed algorithm “fastIsoboles,” which extends one‐dimensional binary search to finding arbitrary curves in two dimensions. The effective isobole (i.e., all dose combinations achieving the efficacy target), is shown as the green curve. (h) Classification of dose combinations into efficacious or nonefficacious solely based on the information contained in the effective isobole. (i) Distribution of effective isoboles resulting from parameter uncertainty. (j) Confidence level response surface resulting from aggregating the distribution of effective isoboles with the “aggregateIsoboles” algorithm. The highly resolved confidence level response surface was obtained at a fraction of the computational cost of the brute force approach

Including parameter uncertainty in a response surface analysis has to overcome two main challenges which are highlighted in the top row of Figure 1. First, to summarize the variability of the predicted efficacy end point due to IIV and parameter uncertainty into an output which is easy to interpret and can be visualized as heatmap. Second, to keep the computing time reasonable.

The first issue is solved by introducing an efficacy target (e.g., reaching 90% treatment success rate, as sketched in Figure 1a,b), where success rate distributions resulting from parameter uncertainty are compared to the efficacy target at different doses. The likelihood of achieving the efficacy target (i.e., the confidence level), can be obtained from the quantiles of the simulated distributions. The confidence level as model output is informative in terms of efficacy as well as parameter uncertainty. For combination therapies, the confidence level response surface could be obtained in a brute‐force manner from simulations at different dose combinations.

The second issue of computational cost is addressed by efficient algorithms. The idea of the procedure is outlined by example of monotherapy in Figure 1c–f and generalized to combination therapy in Figure 1g–j. For each realization of parameters from the uncertainty distribution, the effective dose reaching the efficacy target can be found by line search algorithms, such as bisection^18^ (Figure 1c), as long as the dose‐response curve is monotonously increasing. The effective dose separates the dosing space into efficacious and nonefficacious regions (Figure 1d): checking whether a dose is greater than the effective dose fully informs about clinical success. Due to parameter uncertainty, the effective dose itself follows a distribution, exemplified in Figure 1e. The confidence level is then readily derived from the cumulative distribution function of effective doses. The resolution of the dose‐confidence level relationship in Figure 1f is far greater than in Figure 1b, but was achieved with similar computational effort.

The method of effective doses is generalized to combination therapy with newly developed algorithms. The effective dose itself is replaced by the effective isobole (i.e., the curve connecting all doses achieving the efficacy target, shown in Figure 1g as the green curve). The fastIsoboles algorithm, described below, replaces the bisection method. The monotonicity assumption is generalized by requiring that for each efficacy level, all corresponding dose combinations are connected by a single isobole curve. The effective isobole therefore separates the dosing space into nonefficacious and efficacious dose combinations, highlighted in Figure 1h. Parameter uncertainty implies a distribution of effective isoboles, which is shown in Figure 1i, and the resulting confidence level response surface from the generalization of the cumulative distribution function is shown in Figure 1j. This step is performed by the second new algorithm, aggregateIsoboles, by assessing the fraction of populations for which a dose combination is “above” the respective effective isobole.

Both the brute force and the effective isoboles approach require an NLME PK/PD model to compute success rates and their confidence intervals via population simulation. In this work, we assume model parameters were estimated via maximum likelihood estimation and refer to the asymptotic distribution of estimators as parameter uncertainty distribution, quantifying how well parameters are identified. Due to the mixture of parameter uncertainty and IIV, population simulation involves a two‐step Monte‐Carlo sampling process.^19^ Precise mathematical formulations are detailed in the Supplementary Information Text S1. First, a set of population parameters is drawn from the parameter uncertainty distribution. This set of parameters defines the realization of a population and parametrizes a multivariate distribution of individual parameters. In the next step, *N*_sup_ *_j_*, parameter sets of individual subjects are sampled conditioned on the population parameter realization. This two‐step parameter sampling procedure is repeated for a number of populations, *N*_pop_, generating the population ensemble, the highest level in the hierarchical sampling process. The relevant model outputs for individuals are the PD outputs deciding over treatment success or failure. For a population, the relevant output is the success rate, denoting the fraction of individuals with successful treatment and for the population ensemble, the relevant output in this work is the confidence level of reaching the efficacy target.

### fastIsoboles algorithm

**FIGURE 2:**
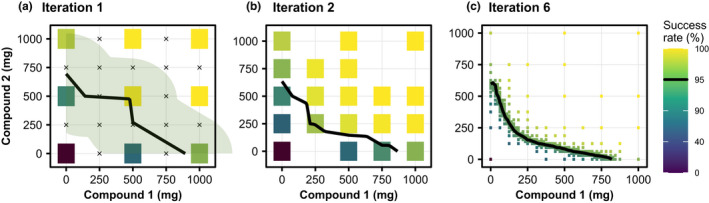
The fastIsoboles algorithm. (a) The algorithm is initialized at nine evenly spaced doses where the objective function is evaluated. The black curve represents the current approximation of the effective isobole of the 95% success rate efficacy target via 2D‐linear interpolation. Dose combinations to be evaluated in the next iteration are indicated by the crosses enclosed by the ribbon. (b) The algorithm at iteration two, with the updated dose combinations and updated approximation of the isobole. (c) The terminated algorithm at iteration six with highly resolved isobole

The fastIsoboles algorithm indicated in Figure 1g is used to efficiently compute the effective isobole of each population. It is a generalization of the bisection method^18^ to two dimensions and is depicted in Figure 2. In contrast to usual optimization or root‐finding algorithms, which converge toward one point in the explored space, this algorithm converges to a whole curve, the effective isobole. The input requirements are the objective function for calculating the success rate *y* = *f* (*AMT*1 and *AMT*2), where *AMTi* denotes the dose of drug i, the efficacy target $\hat{y}$ and the boundaries ${\mathrm{AMTi}}_{\max}$ of the dosing space to be searched. In the example shown in Figure 2, the efficacy target is $\hat{y}=95\text{\%}$ success rate and maximal doses are set to *AMT*1*_max_* = 1000 mg and *AMT*2*_max_* = 1000 mg. The following steps outline the algorithm, a more detailed and mathematically precise description is available in Text S1.

1. The algorithm is initialized by evaluating the success rate at the nine regularly interspaced grid points covering the whole dosing space shown in Figure 2a as colored tiles.
2. The effective isobole is estimated from all evaluated doses by two‐dimensional linear interpolation via the R‐routine *contourLines*.^20^ In Figure 2a, the isobole estimate of the first iteration is depicted as the black line.
3. The grid resolution is doubled, introducing potential new dose combinations to be evaluated shown as crosses in Figure 2a. However, the success rate is only evaluated at a dose combination if the distance between the current estimate of the isobole path and the grid point is smaller than the current grid resolution, saving simulation at uninformative doses. The green ribbon encloses all points evaluated in the next iteration. Figure 2b shows the intermediate result at iteration 2.
4. Repeat steps 2 and 3 until convergence or a predefined maximum number of iterations have been reached. Figure 2c shows the result at iteration 6.

The number of evaluated doses grows exponentially with iteration number. Therefore, only a low number of iterations less than seven is feasible in practice. Seven iterations correspond to a resolution of $2^{7}+1=129$ doses per drug, which will be sufficient in most cases. For the realistic case examples we tested, convergence with respect to the Fréchet distance dF ^21^, ^22^ is demonstrated in Text S1 meaning that the maximum distance of any point on the estimated isobole from the true isobole is given by $dF_{j}\leq \frac{1}{\sqrt{2}2^{j}}$ at iteration number j. Therefore, proximity to the true isobole is guaranteed along the whole estimated isobole by the Fréchet distance metric. As bisection which converges to exactly one root in the interval, fastIsoboles converges to one isobole only, therefore is only applicable to “monotonous” surfaces with one contour line per effect level. An extended discussion of convergence, computational complexity, and limitations is given in Text S1.

### aggregateIsoboles algorithm

**FIGURE 3:**
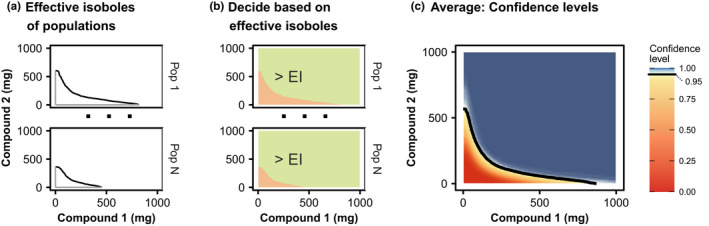
Confidence level response surface. (a) For each population, the effective isobole (EI; black curve) and the segments from the origin up to the intersect of the axes with the isobole (grey) are connected to form a polygon enclosing the dose combinations at which the treatment goal was not achieved. (b) Translation into binary values across all dose combinations. All dose combinations enclosed by the isobole and coordinate axes are shown in red and are coded as zero (for failure: efficacy target is not achieved), all other dose combinations are colored in green and are coded as one (for success: efficacy target is achieved or exceeded). (c) Averaging the binary values from the previous step over all populations for each dose combination results in the confidence level response surface. The 95% confidence level isobole is indicated as black curve

The effective isobole divides the dosing space into two distinct regions indicated in Figure 1h: in the region enclosed by the isobole and the mono‐dose axes, the success rate is below the efficacy target, whereas in the outside region, the success rate is at least the efficacy target. Figure 3 shows how the confidence level response surface is recovered from the distribution of effective isoboles. The steps of the procedure are as follows:

1. Computationally, the effective isobole is represented as an ordered list of points through which it passes. Augmenting this list with the origin of the dosing space yields a polygon enclosing the region in which the efficacy target is not reached, indicated in Figure 3a.
2. For each point of a finely resolved grid, use a fast dedicated algorithm^23^, ^24^ to determine whether the respective dose combination lies outside or within this polygon (i.e., reaches the efficacy target or not). Encode the respective regions with 0 (efficacy target not reached) or 1 (efficacy target achieved or exceeded). In Figure 3b, treatment failure is colored red and success is color coded as green. The recommended resolution of the sampling grid is twice the final resolution of the fastIsoboles algorithm.
3. Repeat steps 1 and 2 for all populations of the population ensemble and average the treatment successes for each grid point. The fraction of populations achieving the efficacy target corresponds to the confidence level of achieving the efficacy target.
4. From the confidence level response surface, isoboles corresponding to different confidence levels can readily be obtained via linear interpolation. The response surface and an exemplary confidence level isobole is visualized in Figure 3c.

In summary, naively extending the methodology of population simulation to combination therapies requires aggregation of simulation results and is impeded by combinatorial complexity. By pre‐specifying an efficacy target, simulations can be summarized by the confidence level of reaching the efficacy target. Simulations can be focused on the most informative dose combinations close to the effective isobole to reduce the computational cost to obtain the full confidence level response surface with a fraction of the simulations compared to the brute‐force approach.

## RESULTS

### Malaria model

#### Description of the PK/PD model and simulations

A current goal in malaria research is the development of novel combination therapies ideally reducing the current 3‐day therapies to a single dose. Administration schemes with single doses rather than the current schemes with repeated doses improve patient compliance and in return are expected to slow down the emergence of resistance due to treatment failures cause by early abandonment of treatment.^25^, ^26^, ^27^ The efficacy target is a cure rate of at least 95% across the population.^27^

An example is shown for malaria where the population PK/PD model is parameterized based on a combination of preclinical and clinical data, and is described in detail including model equations in Text S2. Briefly, plasma concentrations of either drug are described by a two‐compartmental linear elimination model. The parasite population is growing exponentially and killed with a rate depending on the drug concentration with a sigmoidal relationship. Treatment is successful if the parasite concentration in blood is below the limit of quantification of 10 parasites/ml at day 28 after treatment. PD drug‐drug interaction is modeled as empirical Bliss independence model modified to account for different maximal effect sizes, as described by Wicha et al.^8^ For the purpose of this publication, artificial but realistic parameter and covariate values are used. Both drugs’ hypothetical maximum feasible doses are limited to 800 mg.

**FIGURE 4:**
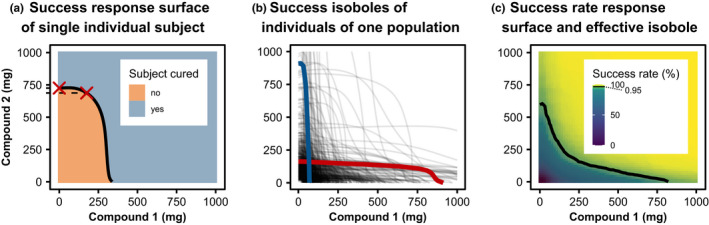
Malaria 1. Individual and population responses of the malaria model. (a) Response surface for one individual and its corresponding isobole (black line). In this case, success corresponds to cure. The black isobole divides the dosing space into unsuccessful and successful doses. At low levels of compound one, the isobole is concave: an increase of compound one dose only slightly decreases the required dose of compound two. (b) All individual cure isoboles for one population: interindividual variability leads to different individual cure isoboles each separating the dosing space into unsuccessful and successful regions differently. In color, are two different patient phenotypes in terms of drug sensitivity to either of the drugs. The blue subject responds well to drug one but poorly to drug two, the red subject vice versa. (c) Success rate response surface for one population and effective isobole (black line) at the target efficacy of 95% success rate. The effective isobole shows all dose combinations that achieve a 95% success rate in that population. The effective isobole is convex: at low doses of compound one, an increase of compound one dose reduces the required compound two dose to maintain the success rate. This beneficial property of the combination therapy is a population level effect

We computed the PD profiles over 28 days after drug administration for 750 populations, each including 1500 subjects; deduced for each individual if treatment was successful or not at day 28 and computed the effective isobole corresponding to the efficacy target of 95% success rate for each of the 750 populations with the fastIsoboles algorithm using six iterations and calculated the confidence level response surface using aggregateIsoboles. For illustration, we simulated one population on the full dose grid to compare the cure isobole of individual patients to the success rate isobole of one population in Figure 4.

#### The difference between individual level and population level isoboles

Despite concave cure isoboles for individual subjects, the success rate isobole of a population can be convex. When starting from the effective mono‐dose of drug two indicated as the red cross in Figure 4a, adding a small amount of drug one does not significantly reduce the amount of drug two required to cure the subject. However, cure isoboles can differ quite drastically between individual subjects as a consequence of IIV. For example, patients can be strong or poor responders for either of the drugs. Examples are given in Figure 4b, where each isobole corresponds to the cure‐isobole of a subject sampled from IIV. A subject with low response to compound two is depicted in blue and a poor responder to compound one is shown in red. On the population level, both subjects push the effective isobole shown in Figure 4c outward only for their respective ineffective drug but not for the other drug. Even though adding small amounts of drug one to the effective mono‐dose of drug two does not help the red subject, on the population level there is a benefit, reducing the required dose of drug two to achieve the target success rate.

#### Characterizing the drug’s dose‐response under uncertainty

**FIGURE 5:**
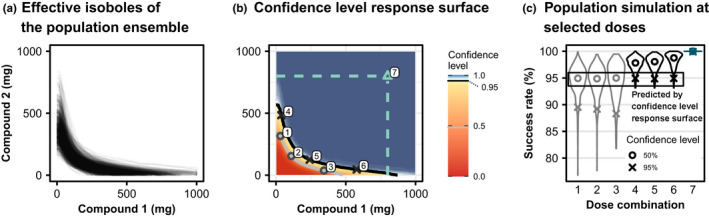
Malaria 2. Effective isoboles and confidence level response surfaces of the malaria model. (a) Different effective isoboles for different realizations of population parameters from the parameter uncertainty distribution. The isoboles of 750 populations, each including 1500 subjects are shown, the efficacy target is a success rate of 95%. (b) The confidence level response surface of achieving at least 95% success rate in the population. The grey isobole corresponds to a 50% confidence level, the black isobole to 95% confidence. Cyan dashed lines indicate the (hypothetical) maximum feasible dose. Numbers indicate the locations at which the exemplary population simulations in panel (c) were performed. (c) Traditional outcome of population simulations with the distribution of success rates across the populations simulated: distributions obtained from population simulations at the doses indicated in panel b. Simulations were performed independently from the calculations of the response surface to validate the results. The circles indicate the median of the distributions, while crosses indicate the lower end of the 95% left‐open confidence interval ranging between the 5% and 100% quantiles. The confidence level response surface indicates at which quantile the target success rate is located, but does not make any other statement about the rest of the distribution of success rates

The confidence level response surface summarizes the influence that parameter uncertainty has on the effective isoboles for the targeted 95% success rate because they indicate the likelihood of reaching the treatment target. Sampling population parameters from their uncertainty distribution leads to different realizations effective isoboles, which are shown in Figure 5a. Using the aggregateIsoboles algorithm, these isoboles were translated into the confidence level response surface shown in Figure 5b. Two confidence level isoboles are shown in Figure 5b as well: the black curve indicates the confidence level isobole of dose combinations that have a 95% probability of reaching the target success rate of 95%, whereas the grey line visualizes the dose combinations having only a 50% chance to do so.

The interpretation of the confidence level response surface is tightly connected to the prespecified efficacy target. At any given dose combination, the probability of achieving the efficacy target is known, but the rest of the success rate distribution is unknown. To emphasize this, population simulations were performed with an independent population ensemble at selected points along the 50% (circles, labels 1–3) and 95% (crosses, labels 4–6) confidence level isoboles which are shown in grey and black in Figure 5b. The respective success rate distributions are shown in Figure 5c as violin plots with the 5% quantile highlighted as crosses and the median as circles. The median of the success rate distributions at dose combinations one to three lies at 95% success rate. This directly translates into a 50% probability of reaching a success rate of 95% or above, as predicted. However, it is not known that they would achieve a success rate of about 90% with a probability of 95% (crosses for combinations 1–3). Analogously, for simulation at doses four to six, it is known that they achieve the efficacy target of 95% with a probability of 95%, but probabilities for other success rate values are unknown.

The 95% confidence level isobole achieves the efficacy target with dramatically lower doses than the respective maximum feasible dose for each compound. The maximum feasible doses for compounds one and two are indicated by the cyan‐colored lines in Figure 5b and population simulation predicts 100% efficacy at the combination of these doses (dose combination 7). The dose combinations enclosed by the maximum feasible doses and the black 95% confidence level isobole represent the treatment opportunity window and can be analyzed further to optimize treatment with regard to cost, safety, and efficacy.

### Antibiotics model

We now apply the methodology to combination therapies of antibiotic treatments. Two antibiotics frequently used in combination to overcome resistance in Gram‐positive methicillin‐resistant staphylococcus aureus (MRSA) bacterial infections are vancomycin^2^, ^28^, ^29^ (VAN) and meropenem^30^, ^31^ (MER). The semimechanistic PD model of VAN‐MER treatment was developed and calibrated on in vitro data of clinical isolates of MRSA.^2^, ^8^ It features the relevant phenomena, such as the Eagle effect,^32^ and the synergistic interaction between the two drugs. In their publication, the authors link this model to previously published PK models^31^, ^33^ in order to perform exemplary clinical trial simulation. The plasma concentrations of VAN and MER serve as direct inputs for the PD model. Simulations were performed for 500 populations, including 1000 subjects each over the time interval of 24 h at a dosing regimen of 2 × 1000 mg VAN b.i.d. and 3 × 1000 mg t.i.d. The clinical end point of interest is a 1000‐fold reduction of bacterial burden within 24 h, denoted as the bactericidal end point. All mathematical equations and details of the model are available in Text S3. The efficacy target is a success rate of 95% of patients meeting the bactericidal end point.

**FIGURE 6:**
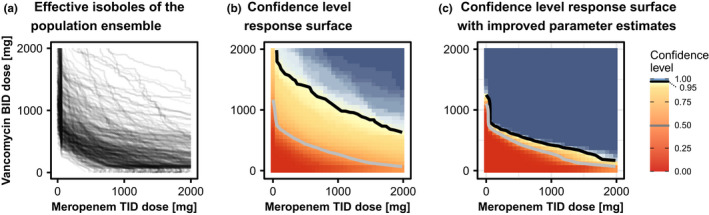
Antibiotics. Effective isoboles and confidence level response surfaces of the antibiotics model. (a) Effective isoboles for antibiotics combination therapy treatment. The target efficacy is a 95% success rate of reaching the bactericidal endpoint. The given parameter uncertainty imposes a large variation between the population realizations. (b) The confidence level response surface of achieving the efficacy target of at least 95% success rate in the population. The grey isobole corresponds to a 50% confidence level, the black isobole to 95% confidence. The two confidence levels’ isoboles are rather distant, indicating the large influence of parameter uncertainty. (c) The standard deviations of the nine most influential parameters were reduced 20‐fold after a hypothetical experiment informing those parameters. The confidence level response surface changed dramatically, allowing for lower doses to be selected with high confidence: With improved parameter estimates, the confidence level of reaching 95% success rates at a dose combination of 1000 mg MER +500 mg VAN is about 99% whereas with poor parameter estimates (panel b), the confidence level at this dose combination is only about 75%. MER, meropenem; VAN, vancomycin

The two main features of the effective isoboles shown in Figure 6a are the strong synergistic effect that MER has on VAN and the large spread of the effective isoboles due to parameter uncertainty. The strong synergism becomes apparent at very low MER doses, which suffice to reduce the amount of VAN required to achieve 95% success and appears to hold for most parameter realizations. Second, the effective isoboles are distributed across the whole scanned dosing space (Figure 6a), which can also be observed in the confidence level response surface with a much broader distribution of confidence levels than in the malaria model (Figure 6b, large yellow and light blue areas). Considering the parameter uncertainty, success rates of 95% are just about achieved with high confidence at the clinically relevant dose of 1000 mg MER t.i.d. and 1000 mg VAN b.i.d.^8^

In this example, the dose assessment is impeded by poorly informed parameters giving rise to the wide distribution of doses that could still meet the treatment target. The isobole simulation results can be analyzed to identify the critical parameters that need to be determined more precisely to gain less uncertain dose predictions, thus aiding experimental design. Correlating the location of the effective isoboles with their corresponding population parameters identifies which parameters influence the predictions most strongly, see Text S3 for more details. This sensitivity analysis reveals which parameters the experimental design should focus to reduce the variability of the prediction most efficiently. In Figure 6c, the standard error of the nine most influential parameters were reduced to 5% of their original value, which could be achieved by additional experimental data for parameter estimation. The spread of the confidence levels is greatly reduced and the new information would allow to confidently reduce the recommended dose amounts without the risk of underdosing.

### Speed improvement

The fast algorithms allowed to calculate both confidence level response surfaces at high resolution. In comparison to the brute‐force method of simulating the population ensemble at each grid point, the total number of simulations was reduced by 21‐fold for the malaria model after six iterations and 6.7‐fold for the antibiotics model after five iterations. The relative saving compared to brute‐force depends on the shape of the effective isobole and increases with the number of iterations. More details on the speed gain and a benchmark of the algorithm’s convergence can be found in Text S1.

## DISCUSSION

Connecting population PK/PD models and response surface analysis leads to new insights into the behavior of combination therapies. Isoboles and response surfaces of individuals, populations, and the population ensemble each carry different insights to understand properties of a given combination therapy. First, the individual‐level PK/PD response surface accounts both for PD interactions as well as the PK properties, which are relevant in the clinical context. As a population effect, it was shown that combinations with concave isoboles for individuals can still improve success rates because IIV allows for realizations of subjects which respond differently well. This is in line with similar findings of Palmer and Sorger in the oncologic setting.^34^ Last, informed decisions need to account for the effects of parameter uncertainty on the predictions which is covered by confidence level response surfaces and isoboles.

Reporting high‐dimensional simulation results intuitively is critical in communicating with collaborators. Confidence level response surfaces and isoboles are effective visualizations displaying prediction uncertainty, which go beyond population simulation at few selected doses. They can be used to intuitively compare combinations with different partner drugs or to assess the impact of certain parameters in a simulation study, informing experimental design, as shown in the section about the antibiotics model.

By focusing on the efficacy level of interest, highly resolved confidence level response surface simulations become feasible. The combinatorial complexity requires trade‐offs between the complexity of the simulation at a given dose level and the number of dose combinations scanned. In current literature, one of the two trade‐offs is currently made. In the first case, population simulation is performed at relatively few doses.^3^, ^8^, ^25^, ^35^ The doses are typically chosen based on current therapy standards and are highly informative about the simulated doses, as confidence levels can be derived for all target success rates. In other cases, simulations scan a larger dose combination range but are only performed for few different parameter sets.^4^, ^25^ These simulations are useful to understand the mechanistic features of the interaction but might overlook the additional effects introduced by parameter variability from IIV and parameter uncertainty. The proposed methods add a third possibility to the tool set of combination therapy simulation, filling the gap the other two approaches left open. A shortcoming is that the algorithm only allows for fixed dosing schedules, requiring multiple runs when dosing schedule variations should be explored. Models of combination therapy are complex to explore and each simulation approach has their benefits and weaknesses. Only combining the methods of all three approaches allows for a comprehensive understanding of the model.

On the algorithmic side, the main assumption is the monotonicity of the dose‐response relationship. Just as bisection might terminate early and return no root at all for nonmonotonous problems, fastIsoboles is prone to “overlook” the effective isobole in nonmonotonous settings. Furthermore, aggregateIsoboles assumes a single isobole per population as input. Both fastIsoboles and aggregateIsoboles could be adapted to account for nonmonotonous surfaces, but we believe the presented algorithms already cover most use‐cases. Nevertheless, monotonicity of success rate response surfaces should be checked by brute‐force simulations for a few populations before running fastIsoboles for the whole population ensemble. The performance gains of the fastIsoboles algorithm varied between six and 16‐fold reduction of simulations (see Text S1), effectively cutting down simulation times from days to hours. A termination criterion improves this efficiency even more by stopping the algorithm when the isobole curve has converged, shortcutting the last and most expensive iterations. The algorithm is robust with regard to nonlinearities of the isobole, such as the kink at low MER doses in the VAN‐MER model. In some extreme cases, the algorithm might have convergence problems, as we detail in Text S1. However, these cases are likely to be of minor practical relevance. Other path‐finding algorithms which effectively follow the isobole, such as an adaption of an integration‐based profile likelihood algorithm,^36^ were tested but dismissed because of inferior performance in this low‐dimensional setting. The assessment of hyperparameters, such as the population size or number of populations, can be performed by varying those numbers and testing the effective isoboles or confidence level isoboles for stationarity.

Future research could be pointed to more than two partner drugs^6^ and toxicity modeling. Combination therapies with more than two drugs can only be analyzed with this method by high‐dimensional cross sections, when one dose is fixed. The methodology can readily be applied to toxicity modeling by simply replacing the dose‐response by a dose‐toxicity relationship. This way, the dose‐response surface can be optimized constrained by the dose‐toxicity response surface as described by Bottino et al.^4^ allowing for a more sophisticated dose constraint than the one used in the malaria example. We hope that the versatility of the presented algorithms and concepts enable and stimulate future research in other disease areas as well.

## CONCLUSION

The presented method joins the methods of population simulation and response surface analysis. On the one hand, the full complexity of the population PK/PD model is considered, including the dynamics of PKs and PDs, IIV, and parameter uncertainty. On the other hand, a general overview of the combination therapy’s behavior at different dose combinations is given by the response surface. This is made feasible by prespecifying a desired success rate, the efficacy target. The confidence level to reach the target success rate is an informative summary statistic both in terms of efficacy as well as uncertainty. The resulting confidence level response surface and its isoboles are easy to interpret clinically: The confidence level isobole defines the minimum doses that reach the target success rate at a given confidence level.

The method was illustrated with two examples for the indication of malaria and antibiotics with a target success rate based on cure. However, it can be applied to any disease indication: for example, to oncology with a target success rate based on survival or 50% tumor size reduction; or to epilepsy with a target success rate based on 50% reduction in seizure frequency. The only prerequisite is to have a model able to predict the clinical outcome from the administered dose combination.

The algorithms fastIsoboles and aggregateIsoboles resolve the trade‐off between computational complexity and dose resolution by focusing on the efficacy levels of interest and minimizing simulations for uninformative doses. The algorithms are publicly available as R‐package in SI and on https://github.com/IntiQuan/populationIsoboles.

## CONFLICT OF INTEREST

The authors declared no competing interests for this work.

## AUTHOR CONTRIBUTIONS

D.L., A.K., and N.G. wrote the manuscript. A.K., D.K., H.S., J.T., and D.L. designed the research. D.L. performed the research. D.L. and V.M. implemented the methods as R‐package.

## Supporting information

Supplementary Material1

Supplementary Material2

Supplementary Material3

Supplementary Material4
