# Supplementary material for: Efficient simulation of clinical target response surfaces
Source: CPT Pharmacometrics Syst Pharmacol. 2022 Mar 11;11(4):512–23. doi: 10.1002/psp4.12779 (PMC9007598; doi:10.1002/psp4.12779)
Supplement: Supplementary file 1 — Supplementary Material1 [file PSP4-11-512-s004.docx]

Efficient simulation of clinical target isoboles – Supplementary text

Table of contents

[1 Population simulation of nonlinear mixed effects PKPD models 1](#_Toc92738639)

[1.1 NLME models 1](#_Toc92738640)

[1.2 Population simulation 1](#_Toc92738641)

[2 fastIsoboles implementation details 2](#_Toc92738642)

[2.1 Detailed steps 2](#_Toc92738643)

[2.2 Computational complexity and convergence 3](#_Toc92738644)

[3 References 6](#_Toc92738645)

# Population simulation of nonlinear mixed effects PKPD models

## NLME models

Nonlinear mixed-effects (NLME) are frequently used for models describing pharmacokinetics (PK) and pharmacodynamics (PD). The defining equations of NLME models are given by the following equations:

$$\begin{matrix} x_{ij} & =g_{j}(p_{i})+\epsilon_{ij} \\ p_{i} & =A_{i}\theta+B_{i}\eta_{i} \\ \epsilon_{ij} & \sim N(0,\sigma^{2}) \\ \eta_{i} & \sim N(0,\Omega^{2}) \end{matrix}$$

Here, the model output $x_{ij}$ denotes model output $j$ for subject $i$ which is composed by the prediction of model $g$ and the residuals $\epsilon$. The parameter vector $p_{i}$ describes the sum of fixed effects $\theta$ and random effects $\eta_{i}$ which are subject-specific. The matrices $A_{i}$ and $B_{i}$ can be used to account for additional individualization of the parameter transformation, such as encoding for covariates or individualized dosings. The random effects $\eta$ are normally distributed with mean zero and variance $\Omega^{2}$, the residuals $\epsilon$ are normally distributed with mean zero and variance $\sigma^{2}$.

The population parameters $(\theta,\Omega)$ of the model are assumed to be estimated by maximum likelihood estimation. Asymptotically, the distribution of the estimators is given by a multivariate normal distribution with true values as $(\theta_{0},\Omega_{0})$ as mean vector and the Fisher Information Matrix as covariance matrix $\Sigma$:

In the main text, we refer to this distribution as parameter uncertainty distribution. In the asymptotic setting, this distribution can be used to deduce confidence intervals of the parameter estimates.

## Population simulation

Population simulation of NLME models can be performed by Monte-Carlo sampling.

1. From the uncertainty distribution of population parameters, draw a population parameter vector for population $k$.
2. Based on the multivariate distribution parameterized by this parameter vector, draw $N_{subj}$ realizations of patient specific parameters. Given below are the equations for a single subject $i$ of this population:

$$\begin{matrix} p_{ik} & =A_{ik}\theta_{k}+B_{ik}\eta_{i} \\ \eta_{ik} & \sim N(0,\Omega_{k}^{2}) \end{matrix}$$

3. Note that for models with covariates such as bodyweight or sex, the corresponding distributions of these covariates also need to be specified.
4. Simulate the model and summarize the output, e.g. by the cure rate, as defined in the main text
5. Repeat steps 1-3 for $N_{pop}$ populations
6. Summarize the results for all populations by descriptive statistics, e.g. Median and 95% Confidence interval.

Population simulation is the underlying technique for clinical trial simulation and the two words are often used synonymously. However, in this work we emphasize distinction between population simulation and trial simulation, because trial simulation can comprehend additional features like multiple arms, drop-out rates etc which are not considered in simple population simulations as described here.

# fastIsoboles implementation details

This section provides further descriptions of the fast isobole algorithm. The algorithm description in the main text pointed out *what* needs to be done in a specific step, in this section, the *why* and the *how* are covered. Furthermore, the algorithm's computational complexity and convergence properties are described.

## Detailed steps

**Definitions** The input requirements are the objective function $y=f(x1,x2)$, where $xi$ are the two independent variables $i$ to be explored, the objective value and the boundaries $xi_{max}$ of the space to be searched. Applied to population PKPD isoboles, $xi$ refers to doses and $f$ encodes a dose-response relationship.

Calculations of Euclidean distance require the definition of a a *normalized grid* to account for differently potent drugs. To this end, the maximum doses $xi_{max}$ are scaled to 1.

$$xi_{sf}=xi_{normalized}=\frac{xi}{xi_{max}}$$

**Step 1** The algorithm is initialized by evaluating the objective function at nine regularly interspaced grid points given by

$$x1,x2\in(0,x1_{max}/2,x1_{max})\times(0,x2_{max}/2,x2_{max}).$$

**Step 2** A more finely rasterized grid is obtained by two-dimensional linear interpolation between the actually calculated grid points. This step is necessary because the contourLines algorithmin R works best with a regular grid as input. As in higher iterations, not all grid points are evaluated, these values need to be imputed by interpolation. Futhermore, interpolating to a finer grid than the current resolution appeared to be beneficial for algorithm convergence. In the implementation, the finely rasterized grid has resolution $res=1/(2^{j}+1)$, with $j=min(6,iter)$ where $iter$ is the current iteration number. The fine grid points along axis $i$ are therefore given by $\{xi_{fg}=l\cdot1/(2^{j}+1)|l\in0,\ldots,(2^{j}+1)\}$.

**Step 3** The isobole curve is estimated from the values of the interpolated grid by linear interpolation via the R-routine *contourLines*. In this step, the main assumption is the monotonicity of the response surface, which produces a single isobole for the effect level . In cases where this is not satisfied, the contourLines algorithm returns a list of isobole estimtes. These are discarded in the current implementation of the algorithm because the downstream analysis of the isoboles requires single isoboles. The isobole curve is represented as ordered list of points .

**Step 4** The actual grid resolution is doubled, introducing new dose combinations to be evaluated. The grid points $xi_{g}$ along axis $i$ are given by $\{xi_{g}=l\cdot1/(2^{iter}+1)|l\in0,\ldots,(2^{iter}+1)\}$. The objective function is only evaluated at dose combinations which are in proximity to the current estimate of the isobole. Proximity is checked on the normalized grid. For each pair of points between the new grid $(x1_{g,sf},x2_{g,sf})$ and the isobole path , it is checked whether the distance is smaller than the half the diagonal of the grid resolution:

$$\mathrm{proximity}:=(x1_{g,sf}-x1_{p,sf})^{2}+(x2_{g,sf}-x2_{p,sf})^{2}<\frac{1}{2}\frac{1}{(2^{iter}+1)^{2}}$$

**Step 5** Repeat steps 2 - 4 until convergence or the maximum number of iterations have been reached. The *maximum number of iterations* should be between five and seven. Due to the exponential complexity of the algorithm, more than seven iterations are typically not feasible. Four iterations were often found too coarse for the impression of a smooth response surface. *Convergence* is currently checked by a simple heuristic: The area enclosed by the isobole and the coordinate axes is measured via rejection sampling as detailed in main text, methods "Efficient computation of confidence level response surfaces from isoboles". In the current implementation, the resolution of the finely rasterized grid for rejection sampling is $res=1/(2^{8}+1)$. If the area does not change significantly between two iterations, e.g. less than 5%, the algorithm assumes convergence. This convergence criterion allows for parts of the isobole compensating each other's area losses or gains and could therefore terminate early. In practice, this has been a minor issue, but the convergence criterion might be updated to a more robust one.

## Computational complexity and convergence

The number of possible function evaluations $N_{tot}$ grows exponentially with iteration number $N_{tot}=(2^{iter}+1)^{2}$. Benchmark simulations show that this exponential growth also holds true for the fast isobole algorithm, as can be seen in Figure 1b. Therefore, the algorithm is of complexity $\mathcal{O}(\exp(i))$. The actually saved number of computations depends on the number of iterations and the length of the isobole curve with respect to the grid dimensions. Longer curves naturally need more function evaluations, as exemplified in Figure 1c. The distribution of required evaluations compared to brute-force is shown for in Figure 2

Figure 1 Computational complexity


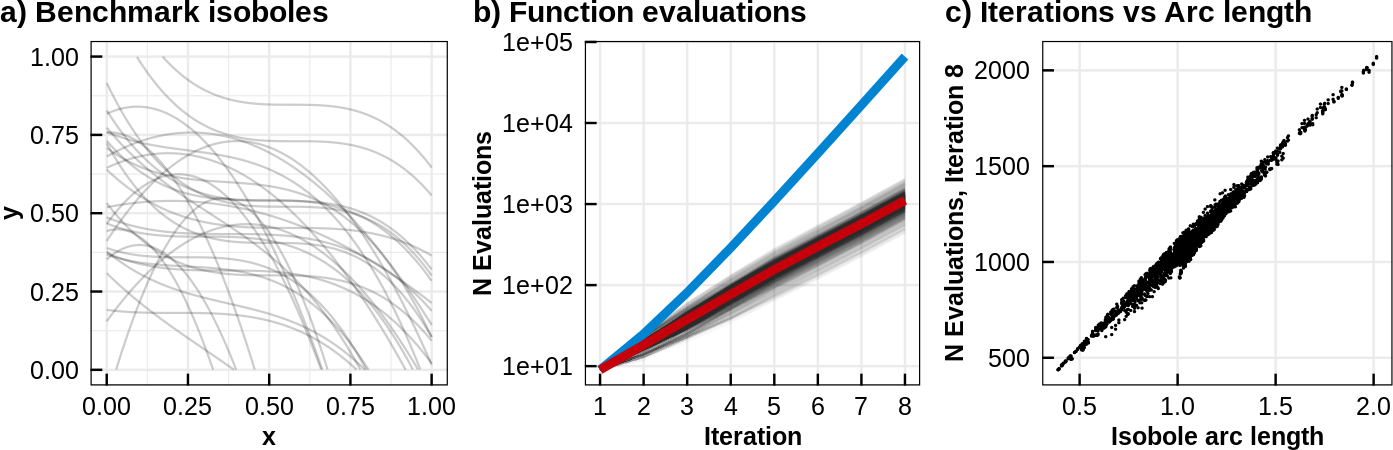


Benchmarking the computational efficiency of the fast isobole algorithm. a) Examples of the benchmark isoboles. b) The maximal number of function evaluations up to iteration *i* are shown in blue. The mean of required iterations by the algorithm is shown in red. Black curves represent individual realizations of benchmark isoboles. c) The number of required function evaluations grows linearly with the isobole's arc length.

Figure 2 Saved iterations


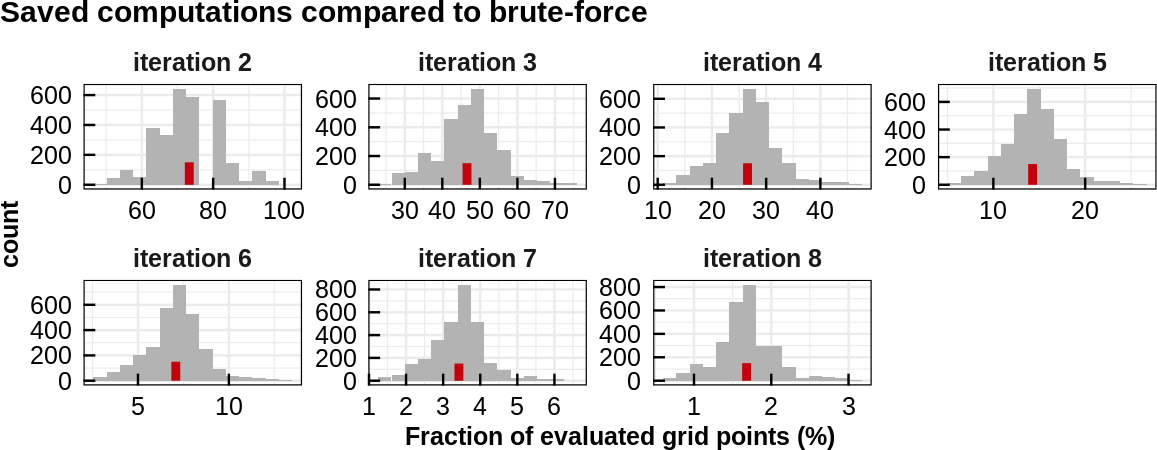


Efficiency of the algorithm on the benchmark isoboles per iteration. The required percentage of function evaluations compared to brute-force decreases with each iteration. The average fraction of required function evaluations is indicated in red.

Convergence of the algorithm can be shown best by the Fréchet distance^1,2^, also known as the "dogleash" metric, depicted in Figure 3a. Even though this criterion did not prove as a useful termination criterion in practice, its theoretical properties make it the perfect metric to assess convergence properties of the algorithm. The Fréchet distance $dF$ between two curves $\gamma_{1}(t_{1}),\gamma_{2}(t_{2})$ is defined by:

$$\begin{matrix} d(t_{1}) & =\min_{t_{2}}(||\gamma_{1}(t_{1})-\gamma_{2}(t_{2})||) \\ dF & =\max_{t_{1}}(d(t_{1})) \end{matrix}$$

Figuratively, for any point on $\gamma_{1}$, the minimal distance $d$ to $\gamma_{2}$ with respect to the $L_{2}$-norm is measured. This corresponds to the blue lines in Figure 3a. This is then summarized by the maximum norm to obtain the Fréchet distance $dF$, shown as green line.

Figure 3 Convergence


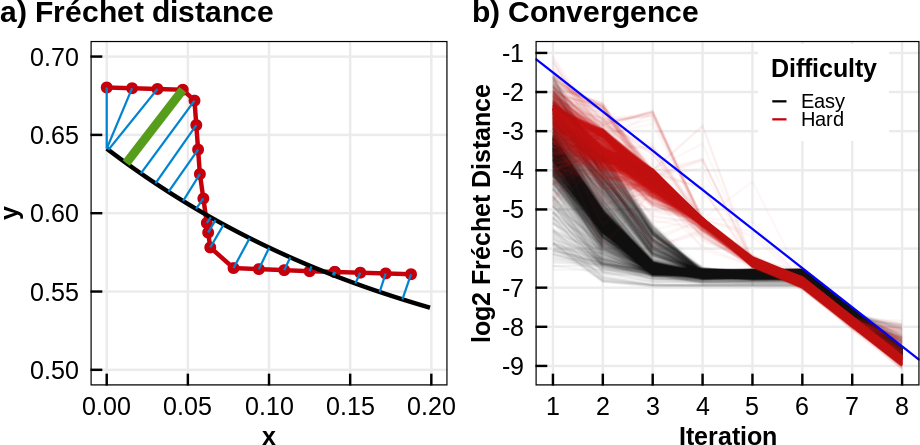


Convergence of the fast isobole algorithm. a) Fréchet distance example. For each point on the red curve, the closest point on the black curve is calculated. The blue lines connect these points. The Fréchet distance between both curves is the length of the green connecting line between both curves, which is the longest minimal distance between the black and the red curve. b) Fréchet distance of isobole estimates at different iterations to true isobole. The black realizations correspond to smoother response surfaces, the red realizations to distorted response surfaces, which are more challenging to the algorithn. The theoretical boundary of Fréchet distance is drawn in blue.

The convergence of the algorithm is assessed by the 3000 benchmark isobole simulations of different shapes and different degrees of non-linearity of the respective response surfaces shown in Figure 1a. The equations underlying the isoboles of the simulation study were as follows:

This way, the true isobole location is defined by the parameters $a,b,c,d$. The distortion of $y$ can be controlled with the parameter $\alpha$. Higher values result in a stronger nonlinear transformation of $y$ values, increasing the difficulty for the algorithm to converge.

In Figure 3b, the Fréchet distance to the true curves is plotted vs. iteration number. The Fréchet distance drops exponentially, therefore the algorithms convergence rate is *linear*:

$$\frac{dF_{iter}}{dF_{iter-1}}=const.$$

The realizations shown in black had a value of $\alpha=1$ which corresponds to a rather linear response surface, resulting in even better convergence due to the interpolation taken in step 2 of the algorithm. Realizations shown in red have the parameter realizations of $a,b,c,d$, but were calculated with $\alpha=5$. In each iteration, the maximum Fréchet distance to the true isobole curve is given by

$$dF_{iter}=\frac{1}{\sqrt{2}2^{iter}}.$$

Some isoboles have trouble converging at lower iteration numbers but converge at higher iterations.

There is a class of functions for which the algorithm *does not converge*, which is exemplified in Figure 4. The objective value is set to . The isobole protrudes into the upper left unit cell "1" of the initial grid but leaves the unit cell before the mid point is reached (Figure 4a). In this case, the four corner points of unit cell 1 are all greater than the objective value and the unit cell is not considered in the next iteration. In fact, the only grid point below the objective value is $(0,0)$ and only points within unit cell 3 are evaluated in iteration 3 as shown in Figure 4b. As the stepsize of the algorithm decreases, the missed information in iteration 2 cannot be compensated for anymore and full convergence cannot be achieved (Figure 4c). This class of functions is probably not of great practical relevance in the application the algorithm was devised for, but if such a case occurs, the algorithm should be re-run with different grid boundaries to more effectively sample the problematic parts of the grid.

Figure 4 Problematic Functions


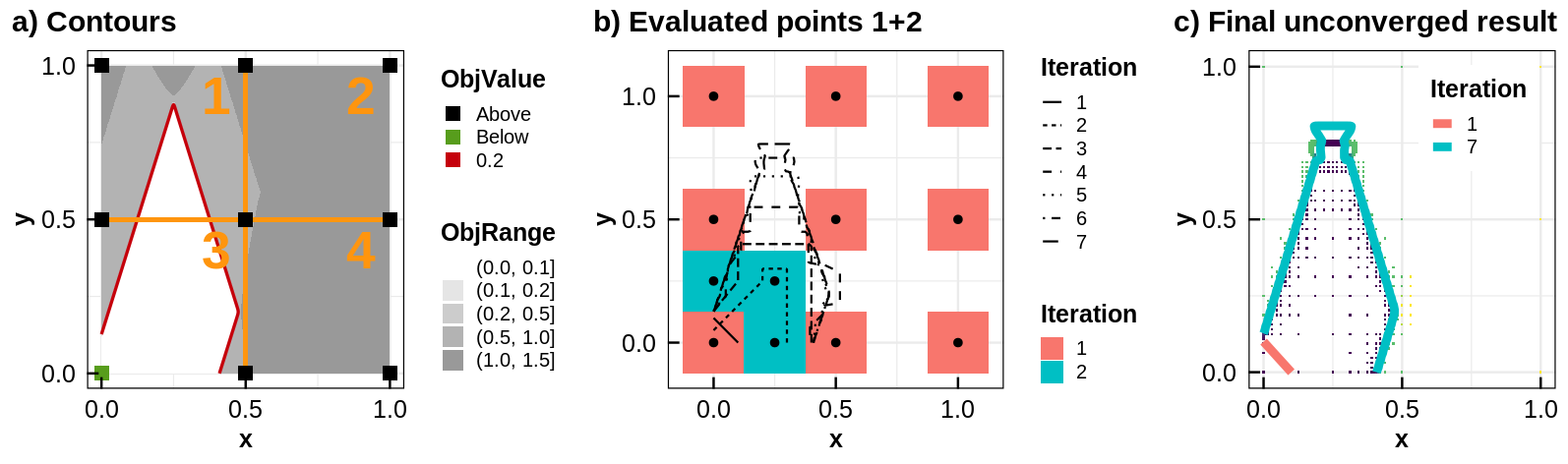


Problematic objective functions. The response surface shows that the objective function is very steep for objective function values between 0.2 and 0.8, as this area is small like a line. The isobole corresponding to y = 0.2 protrudes into unit cell 1, but no proximity is detected. b) The grid points evaluated in the iteration 1 and 2 do not cover unit cell 1. c) The final result shows a bulge in unit cell 1 but is not converged.

# References

1. Fréchet, M. M. Sur quelques points du calcul fonctionnel. *Rendiconti del Circolo Matematico di Palermo (1884-1940)* **22**, 1–72 (1906).

2. Genolini, C. *et al.* kmlShape: an efficient method to cluster longitudinal data (time-series) according to their shapes. *PLOS ONE* **11**, e0150738 (2016).
