# Supplementary material for: Efficient simulation of clinical target response surfaces
Source: CPT Pharmacometrics Syst Pharmacol. 2022 Mar 11;11(4):512–23. doi: 10.1002/psp4.12779 (PMC9007598; doi:10.1002/psp4.12779)
Supplement: Supplementary file 2 — Supplementary Material2 [file PSP4-11-512-s003.docx]

# Malaria Model

## Provided computer code

Executable model R code is distributed as part of the supplementary information SI-Code.zip.

## Malaria model details

All parameter and covariate values were obfuscated, i.e. changed to be unrecognizable and not associated with any real drugs, but still realistic. The interaction parameter $\gamma$ was set to $\gamma=1$. Doses were scaled down to account for lower body weights in children but all results are compared on the scale of adult equivalent doses. Example: A patient's weight is 20 kg, the dose scaling factor is $scale_{dose}=0.5$. In this case, a real dose of $AMT=200\mathrm{mg}$ equals and adult equivalent dose of $AMT_{AE}=400\mathrm{mg}$. The initial parasitemia levels are based on a covariate distribution from data. In the Figures of the main text, all adult equivalent doses are further scaled to have maximum dose 1000mg for each drug.

The final readout is the parasitemia in blood 28 days after treatment. If the parasitemia is predicted to be below the limit of quantification of 10 parasites/mL, the patient is said to be *cured*. Note that this differs from the more commonly used clinical endpoint called *Adequate clinical and parasitological response* (ACPR)^3^, the ACPR additionally includes early and late treatment failures to characterize successful treatment.

### Structural model equations

| **ODEs** | **equation** |
| --- | --- |
| $\frac{d}{dt}Ad_{x1}$ | $-ka_{x1}*Ad_{x1}+Fabs1_{x1}*INPUT1$ |
| $\frac{d}{dt}Ac_{x1}$ | $ka_{x1}*Ad_{x1}-Q1_{x1}/Vc_{x1}*Ac_{x1}+Q1_{x1}/Vp1_{x1}*Ap1_{x1}-CL_{x1}*Cc_{x1}$ |
| $\frac{d}{dt}Ap1_{x1}$ | $Q1_{x1}/Vc_{x1}*Ac_{x1}-Q1_{x1}/Vp1_{x1}*Ap1_{x1}$ |
| $\frac{d}{dt}Ad_{x2}$ | $-ka_{x2}*Ad_{x2}+Fabs1_{x2}*INPUT2$ |
| $\frac{d}{dt}Ac_{x2}$ | $ka_{x2}*Ad_{x2}-Q1_{x2}/Vc_{x2}*Ac_{x2}+Q1_{x2}/Vp1_{x2}*Ap1_{x2}-CL_{x2}*Cc_{x2}$ |
| $\frac{d}{dt}Ap1_{x2}$ | $Q1_{x2}/Vc_{x2}*Ac_{x2}-Q1_{x2}/Vp1_{x2}*Ap1_{x2}$ |
| $\frac{d}{dt}PL$ | $GR-Kkill$ |
| **with** |  |
| $Cc_{x1}$ | $Ac_{x1}/Vc_{x1}$ |
| $Cc_{x2}$ | $Ac_{x2}/Vc_{x2}$ |
| $Eff_{x1}$ | $Cc_{x1}^{hill_{x1}}/(EC{50}_{x1}^{hill_{x1}}+Cc_{x1}^{hill_{x1}})$ |
| $Eff_{x2}$ | $Cc_{x2}^{hill_{x2}}/(EC{50}_{x2}^{hill_{x2}}+Cc_{x2}^{hill_{x2}})$ |
| $Kkill$ | $EMAX_{x1}*Eff_{x1}+EMAX_{x2}*Eff_{x2}-Gamma*Eff_{x1}*Eff_{x2}*min(EMAX_{x1},EMAX_{x2})$ |
| **Initial conditions** |  |
| $Ac_{x1}(t=0)$ | 0 |
| $Ap1_{x1}(t=0)$ | 0 |
| $Ad_{x2}(t=0)$ | 0 |
| $Ac_{x2}(t=0)$ | 0 |
| $Ap1_{x2}(t=0)$ | 0 |
| $PL(t=0)$ | $PLbase$ |

### Parameters and covariate tables

The full table of parameter values including the Fisher Information Matrix can be found in the SI-Code.zip file under SI-Code/01-Models/01-Malaria/Parameters.xls in the “General Parameter Format”, which is documented under https://iqrtools.intiquan.com/doc/book/GPF.html#GPF

Here, we provide the table of population parameter estimates and covariate relationships.

|  |  |  |
| --- | --- | --- |
| **Parameter** | **Value (RSE)** | **Description** |
| Fabs1x1 | 1 (0) | Relative bioavailability Drug1 |
| kax1 | 0.2 (3) | Absorption rate parameter Drug1 |
| CLx1 | 70 (4.5) | Apparent clearance Drug1 |
| Vcx1 | 85 (9) | Apparent central volume Drug1 |
| Q1x1 | 10 (3.5) | Apparent intercompartmental clearance Drug1 |
| Vp1x1 | 1000 (5.5) | Apparent peripheral volume Drug1 |
| Fabs1x2 | 1 (0) | Relative bioavailability Drug2 |
| kax2 | 0.08 (7) | Absorption rate parameter Drug2 |
| CLx2 | 70 (7.5) | Apparent clearance Drug2 |
| Vcx2 | 550 (10) | Apparent central volume Drug2 |
| Q1x2 | 300 (7) | Apparent intercompartmental clearance Drug2 |
| Vp1x2 | 30000 (7) | Apparent peripheral volume Drug2 |
| Tlag1 | 0.4 (1.5) |  |
| Tlag2 | 0.4 (2) |  |
| corr(CLx1,Vcx1) | -2e-04 (100) | IIV Correlation |
| corr(CLx2,Vcx2) | 1 (5) | IIV Correlation |
| beta_kax1(AUC1) | -0.4 (8) | Covariate impact of AUC1 on kax1 via X=X_ref * (AUC1/7.37021451809472)^Beta |
| beta_CLx1(AUC1) | -0.35 (15) | Covariate impact of AUC1 on CLx1 via X=X_ref * (AUC1/7.37021451809472)^Beta |
| beta_CLx1(WT0) | 0.75 (0) | Covariate impact of WT0 on CLx1 via X=X_ref * (WT0/71.2)^Beta |
| beta_Vcx1(WT0) | 1 (0) | Covariate impact of WT0 on Vcx1 via X=X_ref * (WT0/71.2)^Beta |
| beta_Q1x1(WT0) | 0.75 (0) | Covariate impact of WT0 on Q1x1 via X=X_ref * (WT0/71.2)^Beta |
| beta_Vp1x1(WT0) | 1 (0) | Covariate impact of WT0 on Vp1x1 via X=X_ref * (WT0/71.2)^Beta |
| beta_CLx2(WT0) | 0.75 (0) | Covariate impact of WT0 on CLx2 via X=X_ref * (WT0/71.2)^Beta |
| beta_Vcx2(WT0) | 1 (0) | Covariate impact of WT0 on Vcx2 via X=X_ref * (WT0/71.2)^Beta |
| beta_Q1x2(WT0) | 0.75 (0) | Covariate impact of WT0 on Q1x2 via X=X_ref * (WT0/71.2)^Beta |
| beta_Vp1x2(WT0) | 1 (0) | Covariate impact of WT0 on Vp1x2 via X=X_ref * (WT0/71.2)^Beta |
| GR | 0.065 (6) | Net parasite growth rate |
| EMAXx1 | 0.19 (4.5) | Maximum clearance rate |
| EC50x1 | 0.004 (30) | Concentration achieving 50percent of maximum effect |
| hillx1 | 3 (15) | Hill coefficient |
| EMAXx2 | 0.26 (3) | Maximum clearance rate |
| EC50x2 | 0.0085 (20) | Concentration achieving 50percent of maximum effect |
| hillx2 | 6 (15) | Hill coefficient |
| Gamma | 1 (1.5) | Bliss independence interaction parameter |
| omega(kax1) | 0.08 (40) | Random effects |
| omega(CLx1) | 0.35 (8.5) | Random effects |
| omega(Vcx1) | 0.7 (9.5) | Random effects |
| omega(Q1x1) | 0.05 (0) | Random effects |
| omega(Vp1x1) | 0.55 (15) | Random effects |
| omega(kax2) | 0.45 (10) | Random effects |
| omega(CLx2) | 0.65 (8.5) | Random effects |
| omega(Vcx2) | 0.7 (10) | Random effects |
| omega(Q1x2) | 0.6 (9) | Random effects |
| omega(Vp1x2) | 0.6 (9) | Random effects |
| omega(Tlag1) | 0.1 (15) | Random effects |
| omega(Tlag2) | 0.15 (10) | Random effects |
| omega(EMAXx1) | 0.05 (0) | Random effects |
| omega(EC50x1) | 0.3 (0) | Random effects |
| omega(EMAXx2) | 0.05 (0) | Random effects |
| omega(EC50x2) | 0.3 (0) | Random effects |
