# Supplementary material for: Efficient simulation of clinical target response surfaces
Source: CPT Pharmacometrics Syst Pharmacol. 2022 Mar 11;11(4):512–23. doi: 10.1002/psp4.12779 (PMC9007598; doi:10.1002/psp4.12779)
Supplement: Supplementary file 3 — Supplementary Material3 [file PSP4-11-512-s002.docx]

# Antibiotics model

## Computer Code

Executable model R code is distributed as part of the supplementary information SI-Code.zip.

## Model details

The model is described in detail in the original publication^1^. Additional to the original paper, the PK models were simulated considering parameter uncertainty. The uncertainties were taken from the respective original publications. In Li et al.^2^, the standard errors (SE) were given in Table II and transformed to relative standard errors (%) by $RSE(\%)=SE/(\mathrm{PARVALUE})*100$. Here, $\mathrm{PARVALUE}$ refers to the point estimate of the respective parameter. In the paper by Llopis-Salvia et al.^3^, the parameter point estimate value and the 95% confidence interval is reported in Table 3. Assuming a Gaussian distribution, the SE was first extracted via the formula $95CI=4*SE$, where $95CI$ refers to the width of the 95% confidence interval. This formula is based on the fact that roughly 95% of the mass of the Gaussian probability density function lies in the interval of $\mu\pm2\sigma$, where $\mu,\sigma$ denote the mean and standard deviation of the Gaussian distribution. The SE was then used to calculate relative standard errors as described for the example of Li et al.

### Most influential parameters for variability of population isoboles

Each population success rate isoboles is associated with unique population parameters. Unlike in mono-therapy where minimal successful doses can directly be correlated to population parameter values, a different measure for dose has to be found since multiple dose combinations lead to the same success rate. One such a measure is the area enclosed by the isoboles and the mono-dose axes, compared to the full explored grid. An example is visualized in Figure S5a, where about 36% of the explored doses are unsuccessful combinations. Plotting this area against the population parameter realizations for all simulated populations parameters yields Figure S5b. Clearly, some parameters are strongly correlated with the location of the isoboles. The most influential parameters could for example be found by a stepwise regression approach, were the population parameters predict the covered area. This is shown in Fig S5c, where the adjusted R-squared of a forward step regression approach is shown. For example, the linear model *lm(AREA ~VP1_aux_2 + CL2_CR_x3)* explains 50% of the variability of the areas. Consequently, reducing the variance of these parameters in the multivariate uncertainty distribution will therefore reduce the spread of the population isoboles and more spatially confined predictions. The nine most influential parameters for the variability are (in order): Vp1_aux_x2, CL1_CR_x3, CL1_WT_x3, CL_aux_x2, Vc_aux_x2, Q1_aux_x2, CL_omega_x3, Vp1_aux_x3, Vp1_omega_x2. The model equations are given in IQRmodel format in the Supplementary Code. All of the parameters are PK related parameters highlighting the necessity of improving the PK characterization of both drugs.

**Figure S5**


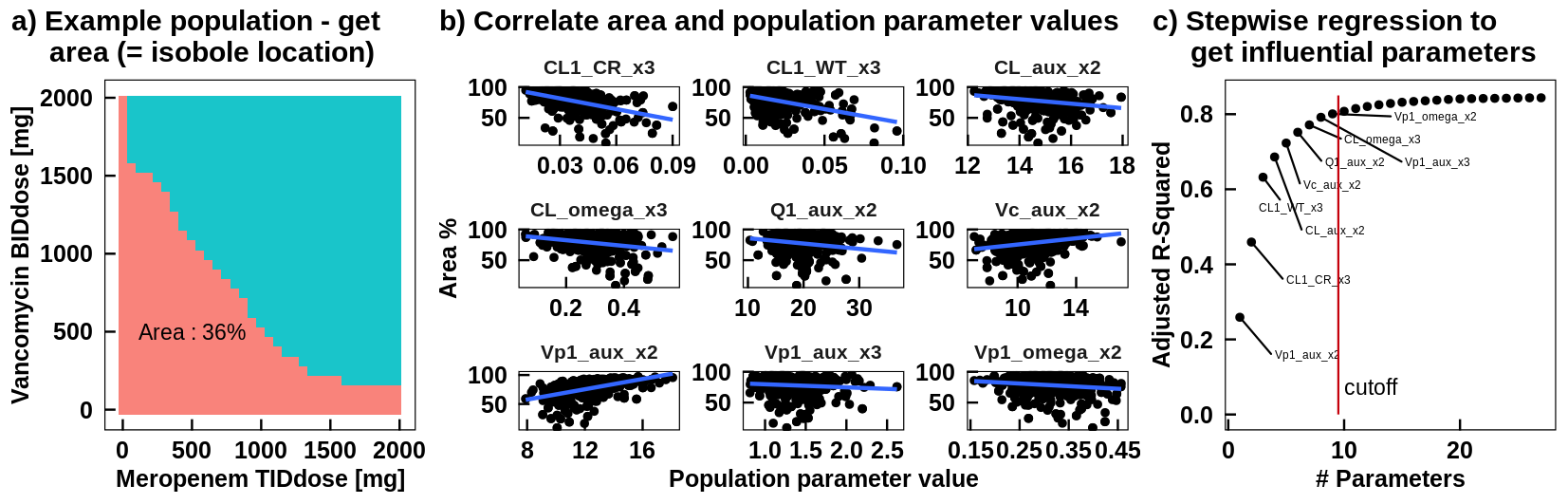


### Structural model equations

| **ODES** | **Equation** |
| --- | --- |
| $\frac{d}{dt}Ad_{x1}$ | $-kabs_{x1}*Ad_{x1}+Fabs1_{x1}*INPUT1$ |
| $\frac{d}{dt}Ac_{x1}$ | $kabs_{x1}*Ad_{x1}-CL_{x1}/Vc_{x1}*Ac_{x1}$ |
| $\frac{d}{dt}Ac_{x2}$ | $-Q1_{x2}/Vc_{x2}*Ac_{x2}+Q1_{x2}/Vp1_{x2}*Ap1_{x2}-CL_{x2}/Vc_{x2}*Ac_{x2}+Fabs0_{x2}*INPUT2$ |
| $\frac{d}{dt}Ap1_{x2}$ | $Q1_{x2}/Vc_{x2}*Ac_{x2}-Q1_{x2}/Vp1_{x2}*Ap1_{x2}$ |
| $\frac{d}{dt}Ac_{x3}$ | $-Q1_{x3}/Vc_{x3}*Ac_{x3}+Q1_{x3}/Vp1_{x3}*Ap1_{x3}-CL_{x3}/Vc_{x3}*Ac_{x3}+Fabs0_{x3}*INPUT3$ |
| $\frac{d}{dt}Ap1_{x3}$ | $Q1_{x3}/Vc_{x3}*Ac_{x3}-Q1_{x3}/Vp1_{x3}*Ap1_{x3}$ |
| $\frac{d}{dt}GRO$ | $-k10*LZD*GRO-k12*(1-LZD)*GRO+k21*(1-MER*(1-MER_{E}agle)*(1-VAN))*(1-Emax_{VAN}*VAN)*REP*2$ |
| $\frac{d}{dt}REP$ | $k12*(1-LZD)*GRO-k21*REP-k{23}_{MER}*MER*REP-k{23}_{VAN}*VAN*REP$ |
| $\frac{d}{dt}PER$ | $k{23}_{MER}*MER*REP+k{23}_{VAN}*VAN*REP-k30*PER$ |
| $\frac{d}{dt}ARoff_{MER}$ | $-(1-VANadaptonMER)*tau_{MER}*cMER_{t}*ARoff_{MER}$ |
| $\frac{d}{dt}ARon_{MER}$ | $(1-VANadaptonMER)*tau_{MER}*cMER_{t}*ARoff_{MER}$ |
| $\frac{d}{dt}ARoff_{VAN}$ | $-tau_{VAN}*cVAN_{t}*ARoff_{VAN}$ |
| $\frac{d}{dt}ARon_{VAN}$ | $tau_{VAN}*cVAN_{t}*ARoff_{VAN}$ |
| **with** |  |
| $CL_{x1}$ | $exp(log(CL_{aux,x1})+CL_{rand}*CL_{omega,x1})$ |
| $Vc_{x1}$ | $exp(log(Vc_{aux,x1})+Vc_{rand}*Vc_{omega,x1})$ |
| $Cc_{x1}$ | $Ac_{x1}/Vc_{x1}$ |
| $CL_{x2}$ | $exp(log(CL_{aux,x2})+CL_{rand}*CL_{omega,x2})$ |
| $Vc_{x2}$ | $exp(log(Vc_{aux,x2})+Vc_{rand}*Vc_{omega,x2})$ |
| $Q1_{x2}$ | $exp(log(Q1_{aux,x2})+Q1_{rand}*Q1_{omega,x2})$ |
| $Vp1_{x2}$ | $exp(log(Vp1_{aux,x2})+Vp1_{rand}*Vp1_{omega,x2})$ |
| $Cc_{x2}$ | $Ac_{x2}/Vc_{x2}$ |
| $CL_{rand,SAFE}$ | $if CL_{rand}\geq-1:CL_{rand}; else: 0$ |
| $CL_{x3}$ | $(CL1_{CR,x3}+CL1_{WT,x3})*(1+CL_{rand,SAFE}*CL_{omega,x3})$ |
| $Vc_{rand,SAFE}$ | $if (Vc_{rand}\geq-1: Vc_{rand} else: 0$ |
| $Vc_{x3}$ | $Vc_{aux,x3}*(1+Vc_{rand,SAFE}*Vc_{omega,x3})$ |
| $Vp1_{rand,SAFE}$ | $if Vp1_{rand}\geq-1: Vp1_{rand} else: 0$ |
| $Vp1_{x3}$ | $Vp1_{aux,x3}*(1+Vp1_{rand,SAFE}*Vp1_{omega,x3})$ |
| $Cc_{x3}$ | $Ac_{x3}/Vc_{x3}$ |
| $cLZD$ | $Cc_{x1}$ |
| $cMER$ | $Cc_{x2}$ |
| $cVAN$ | $Cc_{x3}$ |
| $GRO_{init}$ | ${10}^{(log10N0)}$ |
| $CFU_{M}AX$ | ${10}^{(log10CFUmax)}$ |
| $k12$ | $k_{log}*(1-exp(-mu_{lag}*time))*(1-(GRO+REP+PER)/(CFU_{M}AX))$ |
| $alpha_{MER}$ | $1+betax_{MER}*ARon_{MER}$ |
| $alpha_{VAN}$ | $1+betax_{VAN}*ARon_{VAN}$ |
| $cMER_{t}$ | $cMER*0.98$ |
| $cVAN_{t}$ | $cVAN*0.672$ |
| $cLZD_{t}$ | $cLZD*0.866$ |
| $MER$ | $((1*(cMER_{t}*1)^{H_{MER}})/((alpha_{MER}*EC{50}_{MER})^{H_{MER}}+(cMER_{t}*1)^{H_{MER}}))$ |
| $LZD$ | $((1*cLZD_{t}^{H_{LZD}})/(EC{50}_{LZD}^{H_{LZD}}+cLZD_{t}^{H_{LZD}}))$ |
| $VAN$ | $((1*(cVAN_{t}*1)^{H_{VAN}})/((alpha_{VAN}*EC{50}_{VAN})^{H_{VAN}}+(cVAN_{t}*1)^{H_{VAN}}))$ |
| $VANadaptonMER$ | $((1*cVAN_{t})/(EC{50}_{VANadaptMER}+cVAN_{t}))$ |
| $MER_{E}agle$ | $(Emax_{MER,Eagle}*cMER_{t}^{4})/(EC{50}_{MER,Eagle}^{4}+cMER_{t}^{4})$ |
| $FMER$ | $if cMER\geq MIC_{MER}: 1 else 0$ |
| $FLZD$ | $if cLZD\geq MIC_{LZD}: 1 else 0$ |
| $FVAN$ | $if cVAN\geq MIC_{VAN}: 1 else 0$ |
| $log10CFU_{apparent}$ | $log10(GRO+REP+PER)$ |
| $Ntot$ | $log10CFU_{apparent}$ |
| $OUTPUT1$ | $Ntot$ |
| $Cc_{x1}$ | $1$ |
| $Cc_{x2}$ | $1$ |
| **Initial conditions** |  |
| $Ad_{x1}(t=0)$ | $0$ |
| $Ac_{x1}(t=0)$ | $0$ |
| $Ac_{x2}(t=0)$ | $0$ |
| $Ap1_{x2}(t=0)$ | $0$ |
| $Ac_{x3}(t=0)$ | $0$ |
| $Ap1_{x3}(t=0)$ | $0$ |
| $GRO(t=0)$ | $GRO_{init}$ |
| $REP(t=0)$ | $REP_{init}$ |
| $PER(t=0)$ | $PER_{init}$ |
| $ARoff_{MER}(t=0)$ | $ARoff_{{MER}_{init}}$ |
| $ARon_{MER}(t=0)$ | $ARon_{{MER}_{init}}$ |
| $ARoff_{VAN}(t=0)$ | $ARoff_{{VAN}_{init}}$ |
| $ARon_{VAN}(t=0)$ | $ARon_{{VAN}_{init}}$ |

### Parameters and covariate tables

The table of covariate values can be found in the SI-Code.zip file under 01-Models/02-Antibiotics/Covariates.csv

Parameter values, including those for inter-individual variability, covariate transformations, RSE and uncertainty correlation matrix can be found in IntiQuan’s General Parameter Format under SI-Code/01-Models/02-Antibiotics/Parameters.xls

The General Parameter Format is designed to be human readable. More information on the format can be found here: https://iqrtools.intiquan.com/doc/book/GPF.html#GPF

Here, we provide the table of population parameter estimates

|  |  |  |  |
| --- | --- | --- | --- |
| **Parameter** | **Value (RSE)** | **Description** | |
| kabs_x1 | 1000 (62) | Absorption rate parameter (1/hour) | |
| CL_aux_x1 | 2.85 (5.93) | Apparent clearance (L/hour) | |
| CL_omega_x1 | 0.352 (30.6) | Random effect of CL_x1 | |
| Vc_aux_x1 | 33.6 (4.82) | Apparent central volume (L) | |
| Vc_omega_x1 | 0.308 (35.8) | Random effect of Vc_x1 | |
| beta_CL_aux_x1(CLCR) | 0.618 (15.1) | Covariate impact of CLCR on CL_aux_x1 via X=X_ref * (CLCR/60.9)^Beta | |
| beta_CL_aux_x1(CIR_1) | 0.472 (14.2) | Covariate impact of CIR_1 on CL_aux_x1 via X=X_ref * Beta | |
| beta_Vc_aux_x1(WT) | 1 (0) | Covariate impact of WT on Vc_aux_x1 via X=X_ref * (WT/57.9)^Beta | |
| Fabs0_x2 | 1 (0) | Relative bioavailability (-) | |
| CL_aux_x2 | 14.6 (6.36986301369863) | Apparent clearance (L/hour) | |
| CL_omega_x2 | 0.343511280746353 (5.6) | Random effect of CL_x2 | |
| CL_rand | 0 (0) | Apparent clearance (L/hour) | |
| Vc_aux_x2 | 10.8 (14.537037037037) | Apparent central volume (L) | |
| Vc_omega_x2 | 0.378153408023781 (9.2) | Random effect of Vc_x2 | |
| Vc_rand | 0 (0) | Apparent central volume (L) | |
| Q1_aux_x2 | 18.6 (17.7956989247312) | Apparent intercompartmental clearance (L/hour) | |
| Q1_omega_x2 | 0.53851648071345 (28) | Random effect of Q1_x2 | |
| Q1_rand | 0 (0) | Apparent intercompartmental clearance (L/hour) | |
| Vp1_aux_x2 | 12.6 (13.3333333333333) | Apparent peripheral volume (L) | |
| Vp1_omega_x2 | 0.319374388453426 (17) | Random effect of Vp1_x2 | |
| Vp1_rand | 0 (0) | Apparent peripheral volume (L) | |
| Tk0_x2 | 1 (0) | Absorption time (hours) | |
| Tlag1_x2 | 0 (0) | Absorption lag time (hours) | |
| beta_CL_aux_x2(CLCR) | 0.62 (16.1290322580645) | Covariate impact of CLCR on CL_aux_x2 via X=X_ref * (CLCR/83)^Beta | |
| beta_CL_aux_x2(AGE) | -0.34 (38.2352941176471) | Covariate impact of AGE on CL_aux_x2 via X=X_ref * (AGE/35)^Beta | |
| beta_Vc_aux_x2(WT) | 0.99 (34.3434343434343) | Covariate impact of WT on Vc_aux_x2 via X=X_ref * (WT/70)^Beta | |
| Fabs0_x3 | 1 (0) |  | |
| CL1_CR_x3 | 0.034 (33.8235294117647) |  |  |
| CL1_WT_x3 | 0.015 (66.6666666666667) |  |  |
| CL_omega_x3 | 0.292 (27.8595890410959) | Random effect of CL_x3 | |
| Vc_aux_x3 | 0.414 (6.94444444444444) |  |  |
| Vc_omega_x3 | 0.364 (23.646978021978) | Random effect of Vc_x3 | |
| Q1_x3 | 7.48 (8.88368983957219) |  |  |
| Vp1_aux_x3 | 1.32 (20.2651515151515) |  |  |
| Vp1_omega_x3 | 0.398 (22.0100502512563) | Random effect of Vp1_x3 | |
| Tk0_x3 | 1 (0) |  | |
| Tlag1_x3 | 0 (0) |  | |
| beta_CL1_CR_x3(CLCR) | 1 (0) | Covariate impact of CLCR on CL1_CR_x3 via X=X_ref * CLCR * Beta | |
| beta_CL1_WT_x3(WT) | 1 (0) | Covariate impact of WT on CL1_WT_x3 via X=X_ref * WT * Beta | |
| beta_Vc_aux_x3(WT) | 1 (0) | Covariate impact of WT on Vc_aux_x3 via X=X_ref * WT * Beta | |
| beta_Vp1_aux_x3(WT) | 1 (0) | Covariate impact of WT on Vp1_aux_x3 via X=X_ref * WT * Beta | |
| k10 | 0.101576136256507 (0.0585767505959732) |  |  |
| k21 | 100 (0) |  | |
| k23_MER | 0.106117568163845 (1.145765984877) |  |  |
| k23_VAN | 0.0167752552643773 (0.440529809146503) |  |  |
| k30 | 0.228176159934771 (0.1993411582218) |  |  |
| EC50_MER | 0.0219290710239068 (0.00179214158033214) |  |  |
| H_MER | 3.22633112892813 (4.44211902848341) |  |  |
| betax_MER | 9.53375208546137 (1.67107996486454) |  |  |
| tau_MER | 0.473082668380325 (0.160147908735248) |  |  |
| Emax_MER_Eagle | 0.327583355835067 (0.115004927231309) |  |  |
| EC50_MER_Eagle | 1.35029997438919 (1.09447413762154) |  |  |
| EC50_LZD | 0.676372794638589 (0.570592435206118) |  |  |
| H_LZD | 1.55303619708819 (0.888170927751835) |  |  |
| EC50_VAN | 0.46032896642829 (0.0146199793860863) |  |  |
| Emax_VAN | 0.743217961414823 (0.0266780420137523) |  |  |
| betax_VAN | 3.59859108868659 (1.61695820853148) |  |  |
| tau_VAN | 0.0337291457159727 (0.042989526393885) |  |  |
| EC50_VANadaptMER | 0.388583599813638 (0.098940871458391) |  |  |
| log10CFUmax | 9.42622151307566 (0.0381211919857326) |  |  |
| mu_lag | 0.88381788599107 (2.29024545902939) |  |  |
| k_log | 1.56389581520958 (0.817698843850814) |  |  |
| H_VAN | 20 (0) |  | |
| k_deg_MER | 0.01898 (0) | Calculated in V106 | |
| k_deg_VAN | 0.003898 (0) | Calculated in V106 | |
| MIC_MER | 0.125 (0) |  | |
| MIC_VAN | 1 (0) |  | |
| MIC_LZD | 2 (0) |  | |
| log10N0 | 6 (0.00862303333333333) |  |  |
| REP_init | 0 (0) |  | |
| PER_init | 0 (0) |  | |
| ARoff_MER_init | 1 (0) |  | |
| ARon_MER_init | 0 (0) |  | |
| ARoff_VAN_init | 1 (0) |  | |
| ARon_VAN_init | 0 (0) |  | |
| omega(CL_rand) | 1 (0) | Random effects | |
| omega(Vc_rand) | 1 (0) | Random effects | |
| omega(Q1_rand) | 1 (0) | Random effects | |
| omega(Vp1_rand) | 1 (0) | Random effects | |

## References

1. Wicha, S. G., Huisinga, W. & Kloft, C. Translational pharmacometric evaluation of typical antibiotic broad-spectrum combination therapies against Staphylococcus aureus exploiting in vitro information. *CPT Pharmacometrics Syst Pharmacol* **6**, 512–522 (2017).

2. Li, C., Kuti, J. L., Nightingale, C. H. & Nicolau, D. P. Population pharmacokinetic analysis and dosing regimen optimization of meropenem in adult patients. *J Clin Pharmacol* **46**, 1171–1178 (2006).

3. Llopis-Salvia, P. & Jiménez-Torres, N. V. Population pharmacokinetic parameters of vancomycin in critically ill patients. *J Clin Pharm Ther* **31**, 447–454 (2006).
